# Ligation of the spermatic cord in dogs with a self-locking device of a resorbable polyglycolic based co-polymer – feasibility and long-term follow-up study

**DOI:** 10.1186/1756-0500-7-825

**Published:** 2014-11-20

**Authors:** Odd V Höglund, Jessica Ingman, Fredrik Södersten, Kerstin Hansson, Niklas Borg, Anne-Sofie Lagerstedt

**Affiliations:** Department of Clinical Sciences, Swedish University of Agricultural Sciences, Box 7054, SE-750 07 Uppsala, Sweden; University Animal Hospital, Swedish University of Agricultural Sciences, Box 7040, SE-750 07 Uppsala, Sweden; Department of Biomedical Sciences and Veterinary Public Health, Swedish University of Agricultural Sciences, Box 7028, SE-750 07 Uppsala, Sweden; Radi Medical Systems, Palmbladsgatan 10, SE-754 50 Uppsala, Sweden

**Keywords:** Resorbable medical device, Bioabsorbable, Glycolide, Trimethylene carbonate, Block co-polymer, Castration, Ultrasonography, Replacement, Reduction, Refinement

## Abstract

**Background:**

New surgical techniques are developed to enable a quicker, easier and safer surgery with reduced risk of complications and shortened time needed for recovery. A resorbable device, a self-locking loop, was designed for surgical ligation. The objective of this pilot study was to investigate the feasibility of ligating the spermatic cord with the device, its biocompatibility and long-term resorption in dogs.

**Results:**

The device was made of a block co-polymer (glycolide and trimethylene carbonate), manufactured by injection moulding and consisted of a flexible band running through a case with a locking mechanism. Ten devices were tested for ligation of the spermatic cords in five dogs admitted for routine neutering. The dogs were monitored by physical examination and ultrasonography of the site of ligation, area of spermatic cord and medial iliac lymph nodes regularly until no hyperechoic remnants of the device or acoustic shadowing or local tissue reactions were observed. Haemostasis of the spermatic cords was achieved with the devices. On ultrasonography the devices were seen as hyperechoic structures for 2 months after neutering causing acoustic shadowing for 1 month. The dogs were monitored for 3 – 5 months after surgery. Gradual decrease in echogenicity and final disappearance of the hyperechoic structures suggested resorption. Macroscopic and histological *post mortem* examinations were performed in one dog at 3 months after surgery. *Post mortem* examination showed a tissue reaction of a suture granuloma that was restricted in extent at site of the device.

**Conclusions:**

The results of this pilot study suggest biocompatibility and indicate that ligation of the spermatic cord is feasible with the device.

## Background

Maintaining haemostasis during and after surgery is important. Several different methods exist to prevent bleeding such as energy based methods, metal or polymer clips and ligatures where a traditional suture is tied around the vessel. The use of cable ties (tie-raps) enables a quicker and easier ligation procedure [1–5]. However, they should not be left *in situ* as the non-resorbable material of traditional cable ties may cause pathological reactions such as chronic granulomas and fistulas [6–8]. For the same reasons non-resorbable materials should be avoided for tissue ligation [9–13].

To maintain the surgical advantages of traditional cable ties and avoid the problems associated to the non-resorbable material, a new resorbable self-locking device was developed to ligate blood vessels. Initially the device was made of polydioxanone [14, 15]. However, a need for further development was addressed concerning pliability of the device and consistency of manufacturing results [16]. Attention focussed on the potential use of a block co-polymer of glycolide and trimethylene carbonate (GA and TMC), equivalent to a commonly used suture [17]. The material’s degradation is described [18, 19] and is clinically proven [20]. The mechanical performance of the new device and its degradation over time were tested *in vitro* alongside initial short-term tests *in vivo* in pigs. The results suggested that sufficient strength is retained during healing time of the blood vessels [21]. The possible use of the device for neutering of male dogs was suggested, where the appropriate ligation of the spermatic cord is important to prevent haemorrhage [22].

Biocompatibility [23, 24] of a surgical implant is important to investigate. To be of clinical use, the beneficial effects must be greater than the inflammatory response to the material. All resorbable materials trigger a tissue reaction [20, 23–26]. An inflammatory response may be associated with lymph node enlargement. Few studies have described morphological changes in the regional lymph nodes after implantation of resorbable implants [27, 28].

The objective of this pilot study was to investigate the feasibility of ligating the spermatic cord with the device, its biocompatibility and long-term resorption in dogs.

## Methods

### Animals

In a prospective clinical trial five healthy male dogs admitted for routine neutering were included in the study. Dog number 1 was a research dog kept by the Department of Clinical Sciences at the Swedish University of Agricultural Sciences (SLU), Uppsala, Sweden. At time of surgery the dog was geriatric but his health status was considered acceptable for inclusion in the study. The other four dogs were privately owned, aged <1 to 9 years old (Table 1). An informed consent was obtained from the owners before inclusion of their dog in the study. The Uppsala Animal Ethics Committee, Sweden and Swedish Board of Agriculture approved the study (C 70/12).

**Table 1:** **Characteristics of the five dogs and number of days after surgery when no hyperechoic remnants of the device or acoustic shadowing or local tissue reactions were observed on ultrasound examination, performed monthly**

Dog number	Breed	Age (years)	Bodyweight (kg)	Time (days)	Ultrasound examinations (days after surgery)
1	Beagle	13	16	93	10, 30, 72 , 93
2^e^	Alaskan Husky	0.7	17	92	23, 55, 92
3	Border Collie	9	23	86	30, 57, 86
4^e^	Australian Shepherd	1	24	100	25, 58, 100
5	Airedale Terrier	4	30	147	36, 68, 147
Mean ± standard deviation		5.5 ± 5.3	22 ± 5.7	104 ± 25	

Data in right column is number of days between surgery and each follow-up examination.

2^e^ and 4^e^ were examined 13 and 3 days before surgery, respectively.

### The resorbable device

**Figure 1:**
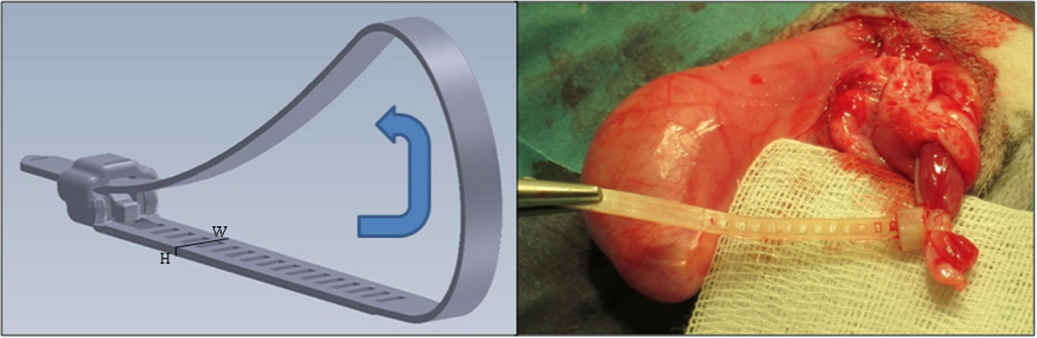
**The design of the device (left) and the device placed around the transected spermatic cord (right).** The width (W) and height (H) of the device indicated in the picture are 4 and 0.65 mm, respectively.

The new device (LigaTie®) was designed for surgery with details enabling complete haemostasis (zero loop diameter) and an enhanced tissue grip (Figure 1). Glycolide (GA) (from BI, CAS 502-97-6) and trimethylene carbonate (TMC) (from BI, CAS 2453-03-4) were polymerized into a block co-polymer. The resorbable polymer was melted and injected into a mould using an in-house built injection moulding machine in a research and development (R&D) laboratory. The devices were placed in aluminium pouches, which were flushed with dry nitrogen gas, and the pouches were sealed. Each pouch contained two devices. Annealing was performed at 65° Celsius for 12 hours. Because a risk of contamination existed during the manufacturing process, the devices were disinfected by soaking them in ethanol 70% for 30 seconds immediately before use.

### Surgery

The surgeries were performed at the University Animal Hospital at SLU. All surgeries were performed by the same surgeon (OVH). The dogs were pre-medicated with sedative and analgesics (acepromazine at 0.3 mg/10 kg i.m., methadone hydrochloride at 2 mg/10 kg s.c. and carprofen at 40 mg/10 kg s.c.). Anaesthesia was induced by i.v. infusion of propofol. Anaesthesia was maintained by inhalation of 2% isoflurane mixed in air. The surgical field was aseptically prepared before surgery, the dogs were placed in dorsal recumbency and the table was tilted with head slightly lower than main body (the Trendelenburg position). An elliptical incision was made at the base of the scrotum. Haemorrhage was controlled by electrocoagulation. The parietal vaginal tunic was incised to expose the testicles (open castration). The spermatic cord (vascular cord and *ductus deferens*) was identified and the flexible band of the device was placed around the spermatic cord. The end of the flexible band was introduced through the locking case, a loop was formed around the spermatic cord and the loop was manually tightened (Figure 1). One device was used for ligation of each spermatic cord. The spermatic cord was transected and inspected for haemorrhage. Excess band at the locking case of the device was removed. The part of the perforated band protruding from the locking case was one perforation in length and the cut-off end was rounded with scissors. The perforation in the protruding band was used to anchor (3–0, poliglecaprone 25, Monocryl, Ethicon) the implant to the subcutaneous tissues at close proximity to the entrance of the inguinal canal, *i.e*. at close proximity to the superficial inguinal ring, without additional encircling of the spermatic cord. The incised fascias and subcutaneous tissues were apposed with resorbable sutures (3–0, poliglecaprone 25, Monocryl, Ethicon). The skin was opposed with an interrupted suture pattern with non-resorbable sutures (nylon, Monosof, Syneture).

### Post surgery and clinical follow-up

Post surgery, the dogs received a collar and stayed for post-operative care and supervision with an additional injection of methadone until they were dismissed later the same day. All dogs were treated with a pain-relieving drug orally (carprofen) at 2 mg/kg twice daily for 7 days. All dogs were later monitored with clinical examination of the surgical area in conjunction with the ultrasound examinations. All dog owners were instructed to monitor their dogs carefully during the study period and contact the surgeon in case of complications.

### Ultrasonography

All dogs were monitored with repeated ultrasonography of the area of the device, the inguinal region’s spermatic cord and the medial iliac lymph nodes. Two dogs were also examined one time each pre surgery. Post surgery study protocol stipulated examinations once every 1 – 2 months until no hyperechoic remnants of the device or acoustic shadowing or local tissue reactions were observed. The surgical area was examined for remnants of the device represented by a hyperechoic structure with or without acoustic shadowing and changes suggestive of a local tissue reaction such as anechoic or echogenic fluid or surrounding hyperechoic tissues. The medial iliac lymph nodes were evaluated for size (length, width and height), shape, contour and echogenicity. The mean (SD) length, width and height of examined lymph nodes were compared between first and second examination after surgery. Pre-surgical measurements were compared to 1 and 2 months after surgery. The level of statistical significance was defined as *p* <0.05 (Student’s t-test, two-sided, paired). The lymph nodes were additionally examined with power Doppler for hilar, peripheral, or mixed hilar and peripheral vascular patterns. The ultrasound examinations were performed on un-sedated dogs with two linear transducers (L9 and L8-18i, LOGIQ E9, GE Healthcare, Milwaukee, Wisconsin 53201, USA) with settings optimized for each patient. All ultrasound examinations were performed by the same radiologist (JI).

### Post mortem examination

Dog number one was euthanized at 3 months after surgery due to geriatric symptoms with weight loss. A *post mortem* examination was performed and samples from the tissue with implant were fixed in 4% phosphate buffered formaldehyde solution for 48 hours. The samples were then further trimmed and multiple samples prepared with 5-mm intervals. The samples were embedded in paraffin, cut in sections (4–5 μm), mounted on glass and stained with hematoxylin and eosin.

## Results

Haemostasis was achieved of all ten ligated spermatic cords and the five dogs were successfully castrated. The dogs showed no signs of complications related to the device during the follow-up period.

### Ultrasound and long-term clinical follow-up

The five dogs were followed regularly for 3 – 5 months post surgery until no hyperechoic remnants of the device or acoustic shadowing or local tissue reactions were observed. In total, 18 ultrasound examinations were performed (Table 1).

Mean (min – max) number of days between surgery and follow-up of all dogs was 29 (23 – 36) days and 62 (55 – 72) days at one and two months, respectively. The third follow-up at three months after surgery was performed at 93 (86 – 100) days for dog number one, two, three and four. The third examination of dog number five was performed 147 days after surgery, which was 79 days after the second examination. Additionally, two dogs (number two and four) were examined 13 and 3 days before surgery and dog number one was examined 10 days post surgery.

No signs of complications related to the devices were found on ultrasound. On ultrasound, the devices were initially hyperechoic and caused acoustic shadowing. A thin distinctly outlined hypoechoic rim of tissue surrounded the devices and was continuous in caudal direction with a hypoechoic slightly heterogeneous oval shaped tissue within each left and right side of the scrotum, presumably representing the pedicles and granulation tissue. The pedicles could not be distinguished from presumed granulation tissue. There was a gradual loss and final disappearance of the echogenicity, distinction and acoustic shadowing of the devices suggesting resorption. The hypoechoic tissues presumed to represent pedicle and granulation tissues were measured in size at every examination and subjectively gradually decreased in size. The spermatic cords cranial to the devices were isoechoic with surrounding tissues and could only faintly be distinguished. The devices were visible for a mean of 2.1 (±0.3) months as hyperechoic structures and caused acoustic shadowing for 1.0 (±0.0) month. The mean time until no hyperechoic remnants of the device or acoustic shadowing or local tissue reactions were observed was 3.6 (±0.9) months.

The lymph nodes were fusiform with clearly defined, smooth margins. The echogenicity was hypo- or isoechoic to surrounding tissues and in 6 of 18 examinations a hyperechoic linear hilus could be seen in one or both lymph nodes.

The mean length, width and height of the lymph nodes at one and two months were 19.5 (±3.7), 6.8 (±2.4) and 5.3 (±1.9) mm versus 21.1 (±4.1), 6.2 (±2.2) and 4.9 (±1.4) mm (*p* =0.13, 1.00 and 0.44, respectively). In 16 examinations power Doppler flow with a hilar vascular pattern was detected in the medial iliac lymph nodes on one or both sides and at 3 examinations vascular flow was not detected in either lymph node.

Overall, the medial iliac lymph nodes were considered normal in size, shape, contour, echogenicity and vascularity at all examinations [29]. In the two dogs examined before surgery there was an increased width of the lymph nodes at one month follow-up, 5.5 (±1) versus 7.5 (±1) mm (*p* =0.04) and there was a tendency for increased length of the lymph nodes at two months after surgery, 19.2 (±3.6) versus 23.2 (±4.7) mm (*p* =0.07).

Dog number one had a small amount (rectangular shaped areas up to 7 mm × 5 mm) of anechoic fluid focally around the left spermatic cord in the inguinal region at 1, 2.5 and 3 months post surgery and in the right inguinal region at 2.5 months post surgery. In the same dog a similar amount (triangular shaped area 5.5 mm × 6.5 mm × 7.5 mm) of fluid was detected at the left implant at 3 months post surgery. In this dog a small amount of fluid was detected within the caudal abdomen (rectangular area 15 mm × 5 mm dorsal to the urinary bladder) at ultrasound 3 months post surgery. Fluid was not found in any of the other dogs.

An extra examination of dog number three and four, outside stipulated study protocol, was performed 133 and 351 days after surgery, respectively, in conjunction with a visit at the animal hospital for reasons not associated to this study. No remnants of device or sutures were seen on ultrasound and size of the lymph nodes was normal.

### Post mortem examination

**Figure 2:**
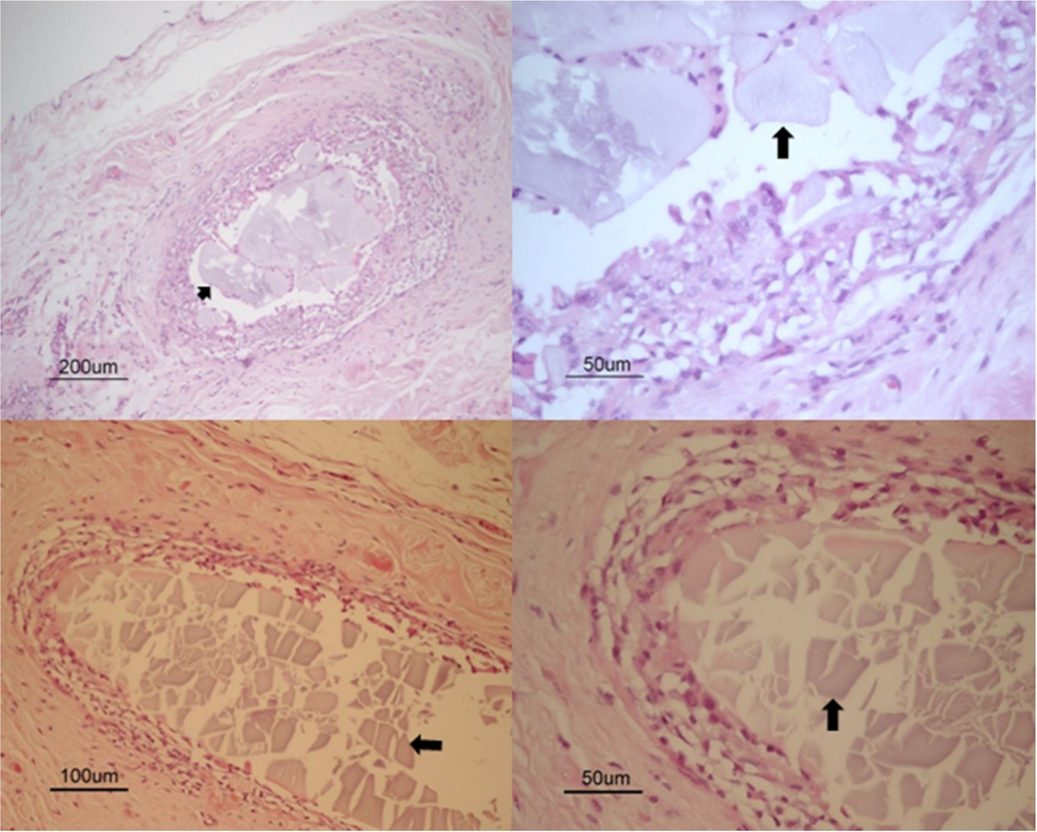
**Remnants of device at centre of images, indicated by arrows.** Close-up of tissue reaction (both right) represented by macrophages and lymphocytes covered by fibroblasts and connective tissue around remnants of device (arrow).

On histological examination of tissue at site of device remnants of material were identified with macrophages, lymphocytes, few multinucleated giant cells and connective tissue. The tissue reaction was restricted in extent. The macrophages were at close proximity to remnants of material and were covered by lymphocytes and fibroblasts, surrounded by matured connective tissue, consistent with a suture granuloma (Figure 2).

At macroscopic examination the lymph nodes were normal with a non-reactive appearance on histological examination. At microscopic examination a mild histiocytosis was observed where some histiocytes were loaded with hemosiderin and others were loaded with different content. At histological examination of the liver a cholangiocarcinoma was diagnosed.

### Miscellaneous complications

Dog number two had loose stools three days after surgery, the carprofen dosage was reduced to half and the problem resolved. Dog number four removed its collar and consequently its skin sutures 5 days after surgery. The skin of the wound broke open, but the subcutaneous tissues were intact. The dog was re-anaesthetized, the wound was cleaned, re-sutured and antibiotics (amoxicillin) were prescribed at 14 mg/kg twice daily for 8 days.

## Discussion

In this study ligation and haemostasis of the spermatic cord was achieved with the device in both medium sized and large breed dogs. As expected, the material of the device was resorbed [20]. The tissue response was in agreement with the natural response after implantation of a resorbable medical device, a foreign body reaction of macrophages, lymphocytes and few multinucleated giant cells, restricted in extent by connective tissue [25]. The results confirmed that sufficient strength of the device was retained during healing time of the blood vessels [21] and allowed adequate time for the inflammatory and proliferative phases of wound healing of the ligated tissue [30]. The results of this pilot study suggest biocompatibility [23, 24], however further studies are warranted.

There is no precise definition or accurate measurement of biocompatibility [31]. Originally the term biocompatibility referred to the ability of a *material* to perform with an appropriate host response in a specific application [23]. Selection criteria for biomaterials therefore evolved as a list of events that had to be avoided. However, some applications required the material to interact with the tissue. Similarly, some applications required the material to degrade over time [32]. The term was later redefined by Williams as “Biocompatibility refers to the ability of a biomaterial to perform its desired function with respect to a medical therapy, without eliciting any undesirable local or systemic effects in the recipient or beneficiary of that therapy, but generating the most appropriate beneficial cellular or tissue response in that specific situation, and optimising the clinically relevant performance of that therapy.” Biocompatibility of an implantable *medical device* can be defined in terms of the success of that device in fulfilling its intended function [24].

Subjectively, the present material was more suitable for injection moulding compared to the previously used polydioxanone [16] because the surface of the product’s flexible band was consistently smooth on macroscopic examination and injection moulding cycles were shortened [21] compared to the previous study [16]. The raw material was pure and the devices could be considered sterile after exposure to high temperature at injection moulding [33]. However, there was a risk of contamination in the later processing of the devices and sterility could not be guaranteed in the used R&D setting. Therefore, as a matter of precaution, the devices were disinfected in ethanol immediately before use. As they were allowed to dry before use, it was unlikely that the ethanol treatment affected the tissue response.

On follow-up examinations after surgery no complications related to the devices were identified. The devices or remnants of the devices were found in their expected place on ultrasound examinations. Resorption of the devices was suggested by the gradual decrease of echogenicity and acoustic shadowing. This was confirmed *post mortem* in one dog where the tissue response was restricted in extent on histology and represented that of a suture granuloma. Resorption of the devices was expected as the material was regarded as equivalent to the material of a commonly used suture which was introduced in the mid 1980s (Maxon™, Syneture, USA) [17].

In a study where polylactide intramedullary implants were used in rabbits, a moderate histiocytosis was found in efferent lymph nodes [27], compared to a mild histiocytosis in the present study. Swollen lymph nodes and active sinus histiocytosis was found following transcortical implantation of polylactide implants in a goat model [28]. In addition to potential differences between animal models, the results are likely affected by the differences in material between the present and previous study, different processes, differences in relative bulk volume of implanted biomaterial, differences in resorption time and timing of *post mortem* examination.

Acoustic shadowing and hyperechogenicity occurred due to expected differences in acoustic impedance between introduced biomaterial and normal body tissues. With time, as the implant degraded, the difference in impedance between tissue and implant decreased. In this study acoustic shadowing distal to the device was seen at one month post surgery. In a previous study [16] a device with an identical design, but of different polymer material, caused acoustic shadowing for a mean of 2.9 months post surgery. In both the present and the previous study [16] implants were seen as hyperechoic structures for about 2 months. The structures examined in this study were anatomically more superficial and higher frequency transducers (up to 18 MHz) were used, enabling better spatial resolution, which may explain the difference in acoustic shadowing. The ultrasound machine used in the previous study [16] did not have spatial compounding technique, and there was a difference between materials with an expected shorter resorption time with the present material. We hypothesize that the main reason for the shorter duration of acoustic shadowing in this study was due to the difference in spatial compounding technique between the ultrasound machines. Acoustic shadowing is suppressed by use of spatial compounding imaging technique [34]. Therefore, in evaluation of resorption time of surgical implants by use of ultrasonography, comparison of acoustic shadowing should be done cautiously if spatial compounding differs between different ultrasound machines. The results of this and a previous study showed that it was possible, to a reasonable extent, to follow the gradual resorption of a resorbable implant with ultrasonography [16]. The use of imaging may reduce the number of animals needed in clinical tests of resorbable devises, which is important for ethical reasons [35].

The increase of width of medial iliac lymph nodes at one month compared to before surgery was transient and the size of the lymph nodes was still within the reference limits [29]. Comparisons to pre-surgical data should be done cautiously because this was restricted to two animals, which is a study limitation. However, reference material for iliac lymph nodes is available [29] and in the present study they were considered normal in size, shape and echogenicity at all examinations which suggest biocompatibility. As the study involved privately owned dogs, there was a variation in follow-up intervals which is an additional study limitation. Another study limitation was the small size of the test group. No controls were used as this study was restricted to analysis of feasibility (proof of concept) of ligating the spermatic cord with the device, its biocompatibility and long-term resorption. Further controlled randomised studies are needed to evaluate potential clinical benefits of the device compared to other surgical techniques. Additional studies may add information regarding resorption of the implant. However, the material used is well known and clinically proven since decades [18–20]. Hemosiderin was found on *post mortem* histological examination of lymph nodes, probably as a consequence to minor haemorrhage at surgery. Dog number one was euthanized due to weight loss in combination with old age. The small amount of fluid seen surrounding the spermatic cords in the inguinal region of this dog may have been situated within the *cavum vaginale* and therefore have originated from the intra-abdominal fluid detected with ultrasound at 3 months post surgery. Gradual loss of echogenicity of implant in dog number one followed a similar pattern as in the other dogs and histopathology showed a normal suture granuloma. This implies the fluid was not caused by the studied device but was instead most likely caused by the liver pathology.

## Conclusion

This pilot study indicates that ligation and effective haemostasis of the canine spermatic cord is feasible with the new resorbable device. The subcutaneous tissue can resorb the quantity of resorbable polymers constituted by the device, which suggests biocompatibility of the device.

## References

[1] Whitney GD (1982). Use of implanted nylon bands in surgical procedures. Canine Pract.

[2] Carpenter RH (1973). Nylon bands used as ligatures and fixation devices in small animal surgery. 40th Ann Meet Am Anim Hosp Assoc.

[3] Zagraniski MJ (1979). Splenectomy using nylon cable tie bands. Feline Pract.

[4] Gofrit ON, Harlev M, Rosenberg S, Pode D, Zorn KC, Shalhav AL, Zamir G, Mintz Y (2010). Pure “cable-tie partial nephrectomy”: a porcine model. Surg Endosc.

[5] Cadeddu JA, Corwin TS, Traxer O, Collick C, Saboorian HH, Pearle MS (2001). Hemostatic laparoscopic partial nephrectomy: cable-tie compression. Urology.

[6] Macedo AS, Dal-Bo ID, de Quadros AM, Brambatti G, dos Reis KDHL, Brun MV, Alievi MM, Beck CAD (2012). Complications associated with ovariohysterectomy using nylon tie-rap as an hemostatic method. Acta Sci Vet.

[7] Johnson-Neitman JL, Bahr RJ, Broaddus KD (2006). Fistula formation secondary to a nylon cable band in a dog. Vet Radiol Ultrasound.

[8] Werner RE, Straughan AJ, Vezin D (1992). Nylon cable band reactions in ovariohysterectomized bitches. J Am Vet Med Assoc.

[9] Pearson H (1970). Ovario-hysterectomy in the bitch. Vet Rec.

[10] Cawley AJ, Archibald J (1958). Sinus tracts resulting from suture material. Can J Comp Med Vet Sci.

[11] Borthwick R (1972). Unilateral hydronephrosis in a spayed bitch. Vet Rec.

[12] Joshua JO (1965). The spaying of bitches. Vet Rec.

[13] Pearson H (1973). The complications of ovariohysterectomy in the bitch. J Small Anim Pract.

[14] Höglund OV (2012). PhD Thesis: A Resorbable Device for Ligation of Blood Vessels. Development, Assessment of Surgical Procedures and Clinical Evaluation.

[15] Höglund OV, Hagman R, Olsson K, Mindemark J, Lagerstedt AS (2011). A new resorbable device for ligation of blood vessels – a pilot study. Acta Vet Scand.

[16] Höglund OV, Hagman R, Olsson K, Carlsson C, Södersten F, Lagerstedt AS (2013). Ligation of the ovarian pedicles in dogs with a resorbable self-locking device – a long-term follow-up study. J Biomater Appl.

[17] Katz AR, Mukherjee DP, Kaganov AL, Gordon S (1985). A new synthetic monofilament absorbable suture made from polytrimethylene carbonate. Surg Gynecol Obstet.

[18] Farrar DF, Gillson RK (2002). Hydrolytic degradation of polyglyconate B: the relationship between degradation time, strength and molecular weight. Biomaterials.

[19] Hill SP, Montes de Oca H, Klein PG, Ward IM, Rose J, Farrar D (2006). Dynamic mechanical studies of hydrolytic degradation in isotropic and oriented Maxon B. Biomaterials.

[20] Pillai CK, Sharma CP (2010). Review paper: absorbable polymeric surgical sutures: chemistry, production, properties, biodegradability, and performance. J Biomater Appl.

[21] Aminlashgari N, Höglund OV, Borg N, Hakkarainen M (2013). Degradation profile and preliminary clinical testing of a resorbable device for ligation of blood vessels. Acta Biomater.

[22] Howe LM (2006). Surgical methods of contraception and sterilization. Theriogenology.

[23] Williams DF (1987). Definitions in Biomaterials. Consensus Conference of the European Society for Biomaterials.

[24] Williams DF (2008). On the mechanisms of biocompatibility. Biomaterials.

[25] Anderson JM, Rodriguez A, Chang DT (2008). Foreign body reaction to biomaterials. Semin Immunol.

[26] Shive MS, Anderson JM (1997). Biodegradation and biocompatibility of PLA and PLGA microspheres. Adv Drug Deliv Rev.

[27] Bondarenko A, Hewicker-Trautwein M, Erdmann N, Angrisani N, Reifenrath J, Meyer-Lindenberg A (2011). Comparison of morphological changes in efferent lymph nodes after implantation of resorbable and non-resorbable implants in rabbits. Biomed Eng Online.

[28] Verheyen CC, de Wijn JR, van Blitterswijk CA, Rozing PM, de Groot K (1993). Examination of efferent lymph nodes after 2 years of transcortical implantation of poly(L-lactide) containing plugs: a case report. J Biomed Mater Res.

[29] Mayer MN, Lawson JA, Silver TI (2010). Sonographic characteristics of presumptively normal canine medial iliac and superficial inguinal lymph nodes. Vet Radiol Ultrasound.

[30] Cornell K, Tobias KM, Johnston SA (2012). Wound Healing. Veterinary Surgery, Small Animal. Volume 1.

[31] Ratner BD, Hoffman AS, Schoen FJ, Lemons JE, Ratner BD, Hoffman AS, Schoen FJ, Lemons JE (2004). Biomaterials Science: A Multidisciplinary Endeavour. Biomaterials Science an Introduction to Materials in Medicine.

[32] W E, Sikalidis C (2011). Biocompatibility. Advances in Ceramics - Electric and Magnetic Ceramics, Bioceramics, Ceramics and Environment.

[33] Konig C, Ruffieux K, Wintermantel E, Blaser J (1997). Autosterilization of biodegradable implants by injection molding process. J Biomed Mater Res.

[34] Heng HG, Widmer WR (2010). Appearance of common ultrasound artifacts in conventional vs. spatial compound imaging. Vet Radiol Ultrasound.

[35] Russell WMS, Burch RL (1959). The Principles of Humane Experimental Technique.

